# Successful selection of rainbow trout (*Oncorhynchus mykiss*) on their ability to grow with a diet completely devoid of fishmeal and fish oil, and correlated changes in nutritional traits

**DOI:** 10.1371/journal.pone.0186705

**Published:** 2017-10-23

**Authors:** Thérèse Callet, Françoise Médale, Laurence Larroquet, Anne Surget, Pierre Aguirre, Thierry Kerneis, Laurent Labbé, Edwige Quillet, Inge Geurden, Sandrine Skiba-Cassy, Mathilde Dupont-Nivet

**Affiliations:** 1 UMR GABI, INRA, AgroParisTech, Université Paris-Saclay, Jouy-en-Josas, France; 2 UMR NuMéA, INRA, St-Pée-sur-Nivelle, France; 3 PEIMA, INRA, Sizun, France; Department of Bionanotechnology, MEXICO

## Abstract

In the context of limited marine resources, the exponential growth of aquaculture requires the substitution of fish oil and fishmeal, the traditional components of fish feeds by terrestrial plant ingredients. High levels of such substitution are known to negatively impact fish performance such as growth and survival in rainbow trout (*Oncorhynchus mykiss*) as in other salmonids. In this respect, genetic selection is a key enabler for improving those performances and hence for the further sustainable development of aquaculture. We selected a rainbow trout line over three generations for its ability to survive and grow on a 100% plant-based diet devoid of both fish oil and fishmeal (V diet) from the very first meal. In the present study, we compared the control line and the selected line after 3 generations of selection, both fed either the V diet or a marine resources-based diet (M diet). The objective of the study was to assess the efficiency of selection and the consequences on various correlated nutritional traits: feed intake, feed efficiency, digestibility, composition of whole fish, nutrient retention and fatty acid (FA) profile. We demonstrated that the genetic variability present in our rainbow trout population can be selected to improve survival and growth. The major result of the study is that after only three generations of selection, selected fish fed the V diet grew at the same rate as the control line fed the M diet, whilst the relative reduction of body weight was 36.8% before the selection. This enhanced performance on the V diet seems to be mostly linked to a higher feed intake for the selected fish.

## Introduction

Aquaculture is one of the fastest growing animal production sectors. Its expansion is a mixed blessing as it allows for the provision of half of all fish consumed by humans worldwide, but significantly increases the need for fishmeal (FM) and fish oil (FO), traditional components of aquaculture feed [1]. This is particularly true for high trophic level fish such as rainbow trout which are large consumers of FO and FM. Aquaculture reliance on both FM and FO must be reduced to enable the further sustainable development of aquaculture, and there are several reasons for this. Fisheries for small pelagic fish highly affect marine ecosystems, and the apparition of fisheries quotas led to the stabilization of FM and FO production [2]. The high demand for FO and FM along with their limited availability affects the supply and demand balance and leads to higher prices, which are no longer viable for aquaculture production [3]. Finally, some of the small pelagic fish used for FM and FO production can be used to feed people [4]. Today, terrestrial plant-based products (vegetable oil, VO and vegetable meal, VM) are some of the most common components used to replace FO and FM due to their high availability and lower cost [5].

The transition towards plant-based diets is highly challenging and the effects of plant ingredients have been extensively studied [6, 7]. Feed formulations have been drastically improved in recent times, allowing substitution rates as high as 80% since first-feeding [8]. Nevertheless, the total substitution of marine ingredients still negatively impacts survival and growth rates in salmonids [9, 10]. Despite being formulated to meet fish nutrient requirements, there exist various differences in nutrient composition between traditional marine resources-based and plant-based diets, which may impact the two main determinants of growth, feed intake and feed efficiency, as well as other nutritional traits. Among these differences, we can point to the fatty acid (FA) profile since plant-based diets are usually richer in omega-6 FA and lack the very-long chained highly unsaturated omega-3 FA (such as EPA or DHA) [11]. Plant-based diets are also characterized by the presence of carbohydrates that are poorly assimilated by carnivorous fish [12], and anti-nutritional factors (ANF) that affect fish feed intake, feed efficiency, metabolism and health [13–15]. Finally, plant-based diets are also known to highly impair feed intake [6, 16].

In parallel to studies which sought to improve diet formulation, others focused on improving the performance of fish fed diets devoid of marine ingredients through genetic selection [17]. Various studies have demonstrated the existence of genetic variability in growth of rainbow trout populations when fed increasing proportions of plant products in feedstuffs [18–21]. Some genotypes seem better able to accept and use these diets than others. Some of these studies have found a significant interaction between diet and genotype for growth [20, 21] as well as for feed intake and feed efficiency [22]. This positive interaction reveals that families selected in previous breeding programs, which perform best with traditional diets, are not necessarily performing their best when fed plant-based diets. These results emphasize the need to develop breeding programs specific to plant-based diets. However, research on these breeding programs are typically focused on FM replacement [23–26] in salmonids.

Thus our team addressed the question of replacement of both FM and FO, and a sequential upward selection was made on the ability to survive and grow with a plant-based diet completely devoid of marine ingredients (both FO and FM) distributed at the very first meal [27]. After only one generation of selection, genetic progress was measured for growth rate, biomass and survival [27]. The objectives of the present study were to assess the efficiency of selection after 3 generations of selection, and to better understand the origins of the progress as well as the consequences of the selection. To answer these questions, we analyzed

1. the efficiency of selection on selected traits: the growth and the survival of rainbow trout
2. the effect of the selection on the correlated nutritional traits: feed efficiency, feed intake, digestibility, composition of whole fish including FA content and nutrient retention.

## Materials and methods

### Selection principles

The base population is the INRA-Synthetic strain (SY), a domesticated strain maintained in the experimental PEIMA facilities (INRA, Sizun, France) fed over generations with a commercial diet, containing a mix of marine and plant ingredients. Breeding is made with a large number of breeders and without artificial selection in order to maintain the genetic variability. From this SY line, a new line (SU) was selected during three generations, for its enhanced ability to survive and grow when fed a plant-based diet totally devoid of fish oil (FO) and fishmeal (FM) (Table 1) [27]. The first generation was issued from a full factorial cross among SY parents. Fish obtained from this cross, were fed from the first-feeding stage with a plant-based diet completely devoid of marine products. The same V-1 diet was used for the first and second generation selections and a different plant-based diet was used for the third (V diet). The two plant-based diets (V-1 and V) were totally devoid of marine products which were replaced by a blend of vegetable meals (wheat, fava bean, corn, soybean, lupin and peas) and vegetable oils (rapeseed, linseed and palm oils)(Table 2). The two plant-based diets were supplemented with amino acids to meet rainbow trout essential amino acid requirements, and with a mineral and vitamin mix.

**Table 1:** Principles of selection for improved survival and grow with a 100% plant-based diet.

Base population	Number of breeders (Sires x Dams)	Initial number of fish	Proportion of selected fish	Diet
**Year 1 (2007): SU-1**				
SY	44 x 32	15000 fish	3.90%	Diet V-1
**Year 3 (2009): SU-2**				
SU-1	45 x 40	10000 fish	4.50%	Diet V-1
**Year 6 (2011): SU-3**				
SU-2	45 x 40	10000 fish	4.90%	Diet V

SY refers to the INRA-Synthetic strain. SU-1, SU-2 and SU-3 refer to the selected line obtained after one, two and three generations of selection, respectively.

**Table 2:** Ingredients, proximal composition of the experimental diets V-1 [27], V, and M, and fatty acid composition of the M and V (2mm) experimental diets (DM: dry matter).

	V-1 diet	V diet	M diet
**Ingrédients** (%)			
**Fishmeal** (Southern hemisphere, Sopropêche, France)	**0**	**0**	**65**
Extruded whole wheat (SudOuest Aliment, France)	0	4	21
Fava bean (CP)	0	10	0
Corn gluten (CP 60; Inzo, France)	25	17	0
Wheat gluten (CP 70; Roquette, France)	23.9	17	0
Soybean meal (CP 48; Inzo, France)	20.8	12	0
White lupin seed meal (Terrena, France)	7	5	0
Extruded peas (Aquatex, Sotexpro, France)	0	12.5	0
**Fish oil** (Southern hemisphere, Sopropêche, France)	**0**	**0**	**11**
Rapeseed oil (Daudruy, France)	6.2	6	0
Linseed oil(Daudruy, France)	3.7	3.6	0
Palm oil(Daudruy, France)	2.4	2.4	0
Soy-lecithin (Louis François, France)	2	2	0
L-Lysine (Eurolysine)	1.5	0.5	0
L-Methionine (Evonik, Germany)	0	0.5	0
CaHPO4.2H20 (18%P; 22%Ca)	3.5	3	0
Min. and Vit. premix, INRA^a^	2	3	3
Attractant mix^b^	0	1.5	0
**Composition (% DM)**			
Dry matter	94.1	96.9	97.6
Crude protein	50.5	51.4	50.1
Crude fat	16.2	18.5	19.4
Starch	-	9.6	14.1
Ash	5.6	6.5	12.7
Energy (kJ/g DM)	23.2	23.6	22.6
**Fatty acid composition (% of total fatty acids)**			
**Saturated**	16.3	19.8	39.2
**MUFA**	40.7	39.0	29.5
**n-6 PUFA**	22.5	24.0	4.7
→ 18:2 n-6 (LA)	22.2	24.0	3.2
**n-3 PUFA**	20.6	17.1	19.3
→ 18:3 n-3 (ALA)	20.6	17.1	1.1
→ 20:5 n-3 (EPA)	0.0	0.0	9.5
→ 22:6 n-3 (DHA)	0.0	0.0	5.2

^*a*^Mineral premix (g or mg kg-1 diet): calcium carbonate (40% Ca), 2.15 g; magnesium oxide (60%Mg), 1.24 g; ferric citrate, 0.2 g; potassium iodide (75%I), 0.4 mg; zinc sulphate (36%Zn), 0.4 g; copper sulphate (25%Cu), 0.3 g; manganese sulphate (33%Mn), 0.3 g; dibasic calcium phosphate (20%Ca, 18%P), 5 g; cobalt sulphate, 2 mg; sodium selenite (30%Se), 3 mg; KCl, 0.9 g; NaCl, 0.4 g (UPAE, INRA); And Vitamin premix (IU or mg kg-1 diet): DL-a tocopherol acetate, 60 IU; sodium menadione bisulphate, 5 mg; retinyl acetate, 15,000 IU; DL-cholecalciferol, 3,000 IU; thiamin, 15 mg; riboflavin, 30 mg; pyridoxine, 15 mg; B12, 0.05 mg; nicotinic acid, 175 mg; folic acid, 500 mg; inositol, 1,000 mg; biotin, 2.5 mg; calcium pantothenate, 50 mg; choline chloride, 2,000 mg (UPAE, INRA).

^*b*^Attractant mix: glucosamine, 0.5 g; taurine, 0.3 g; betaine, 0.3 g; glycine, 0.2 g; alanine, 0.2 g.

Selection was obviously made among the surviving fish, thus survival was the first selected trait. Three to five sortings on fish fork length were carried out between the second and the fourteenth month after the first feeding. The final proportion of selected fish ranged from 3.9% (first generation) to 4.9% (third generation). After the last sorting (14 months), selected fish were fed a commercial diet (B MEGA 20, “Le Gouessant”) to ensure a normal reproduction, and thus prevent any selection on fish reproductive capacity with the V diet. Indeed, Lazzarotto *et al.* [28] showed that rainbow trout females fed all their life with a 100% plant-based diet have very heterogeneous reproductive performances. The commercial diet formulated with a blend of FM, FO and plant ingredients, contains 40% of crude protein and 28% of crude fat. The shift on a commercial diet was made after the last sorting, and this diet should not thus influence the selection. Each new generation was obtained from a full factorial cross between selected fish (at least 44 sires * 32 dams).

### Evaluation of response to selection

To assess the efficiency of selection and its effect on correlated nutritional traits, a 7-month feeding trial was performed on fish issued from the third generation of selection. For each line, the selected (SU) and the control (SY), 3600 eggs were obtained through within line mating (18 dams and 31 sires for each line). Eyed eggs from the 2 lines were randomly distributed into 12 tanks (0.25m^3^) at 19 days post fertilization (dpf), with an average of 600 eyed eggs per tank and maintained at a constant water temperature of 11.4°C, under artificial photoperiod condition (from 8 am to 8pm). From the first feeding (41 dpf) and for a period of 7 months (197 dpf), three batches were fed with a 100% plant-based diet completely devoid of marine products (V diet; SU-V, SY-V). The three other batches for each line were fed with a control marine resources-based diet (M diet; SU-M and SY-M). The V diet was similar as the diet used for the third generation of selection, and the M diet was primarily composed of FM, FO and also contained whole wheat in order to render the diets nearly isoproteic, isolipidic and isoenergetic. Both diets were extruded. Formulation and composition of the two experimental diets are presented in Table 2, along with the fatty acid composition. The V diet was rich in omega-6 long chain FA (n-6 PUFA) due to the presence of linoleic acid (18:2 n-6, LA). While the M diet contained both eicosapentaenoic acid (20:5 n-3, EPA) and docosahexaenoic acid (22:6 n-3, DHA), the V diet was completely devoid of these two fatty acids but contained alpha-linolenic acid (18:3 n-3, ALA), which is the precursor of EPA and DHA. Each diet was distributed automatically over 8 hours throughout the day and rations were adjusted according to biomass for each tank in order to meet satiation. Pellet size evolved during the experiment to adapt to fish body size, but composition remained unchanged.

#### Data collection

The rearing experiment was divided into two distinct periods, the first from the first feeding (41 dpf) to 153 dpf and a second from 153 to 197 dpf. During the first period, it was not possible to accurately measure feed intake because the size of the pellets, adapted to the size of fish mouth, were too small to allow a precise recording of uneaten pellets. During the second period (from 153 to 197 dpf), uneaten pellets were collected daily after each distribution. Estimation of uneaten and consumed quantities allowed for the estimation of the feed intake and feed efficiency.

#### Growth and survival rates

Since 19 dpf, mortality was recorded daily for each tank during the experiment. To monitor growth rate, two or three random samplings of 50 fish in each tank were performed at 61, 78, 99, 118, 133, 153, 174 and 197 dpf.

#### Feed intake and feed efficiency

Daily feed intake (DFI) and feed efficiency (FE) were calculated as follows:

$$\begin{array}{c} DFI=\frac{NI}{{\left(BW_{f}^{0.8}\times BW_{i}^{0.8}\right)}^{0.5}\times 44} \end{array}$$ (1)

$$\begin{array}{c} FE=\frac{BW_{f}-BW_{i}}{NI} \end{array}$$ (2)

with BW_*i*_ and BW_*f*_, the mean body weight (in g wet weight) at the beginning (153 dpf) and at the end (197 dpf) of the period, and NI the net feed intake (in g dry matter (DM)).

#### Whole body composition, nutrient retention and fatty acid profile

In order to analyze the composition of whole fish, nutrient retention, and fatty acid composition, fish were anaesthetized and sampled (10 whole body fish per tank) at the beginning and at the end of the second period (153 and 197 dpf). Fish were individually weighed, frozen and stored at -20°C for further biochemical analyses (detailed below).

Nutrient retention was calculated for X, with X standing for lipids, protein or energy as follows:

$$\begin{array}{c} Retention=\frac{BW_{f}\times X_{f}-BW_{i}\times X_{i}}{NI_{X}}*100 \end{array}$$ (3)

where X_*i*_ and X_*f*_ represent the initial and final carcass content in nutrient (in g) and NI_*X*_ the nutrient X intake (in g DM)

#### Measure of digestibility

At the end of the feeding trial, juveniles were randomly sampled and transferred to the experimental INRA facility of Saint-Pée-sur-Nivelle (France) in order to analyze apparent coefficient of digestibility (ACD). Fish were maintained at a constant temperature of 17°C in a thermoregulated system. Groups of 15 juveniles weighing on average 120 g from the 4 different conditions (SU-M, SU-V, SY-M and SY-V) were maintained in 60L cylindro-conical tanks equipped with an automatic faeces collector (3 tanks per condition). Fish were hand-fed twice a day to visual satiation with either the V or the M diet enriched with 1% Cr2O3, as an inert marker. The faeces were automatically collected over 23 days and stored daily at -20°C for further biochemical analyses (detailed below).

The apparent digestibility were calculated as:

$$\begin{array}{c} AD_{drymatter}=100-\left(100*\frac{Cr2O3_{diet}}{Cr2O3_{faeces}}\right) \end{array}$$ (4)

$$\begin{array}{c} AD_{X}=100-\left(100*\frac{X_{faeces}}{X_{diet}}*\frac{Cr2O3_{diet}}{Cr2O3_{faeces}}\right) \end{array}$$ (5)

where X corresponds to protein, lipid, starch or energy.

#### Biochemical analysis of diets, fish and faeces

Diets, previously reduced to powder, fish samples previously lyophilized after moisture estimation (2 pools of 5 whole fish samples), and faeces previously lyophilized were analyzed. Dry matter content (DM) was measured after drying samples (5g) at 105°C for 24 h. Protein content was estimated by the Kjeldahl method (Nx6.25, Kjeldahl Nitrogen Analyser 2000, Fison Instruments). Gross energy of the samples was measured after combustion in an adiabatic bomb calorimeter. Starch content was measured according to the method described by Thivend et al. [29]. Total lipid extraction was performed according to Folch *et al.* [30], to assess final lipid content.

To analyze the fatty acid (FA) profile, an acid-catalyzed transmethylation was performed from 100 mg of lipids extracted according to Shantha and Ackman [31], to prepare fatty acid methyl esters (FAME). FAME were analyzed with a Varian 3900 gas chromatograph and identified with reference to a known standard mixture (Sigma, St Louis, MO, USA). The results were expressed for each FA as a percentage of total lipids extracted and as quantities (in mg/100g tissue) for both 20:5 n-3 FA (EPA) and 22:6 n-3 FA (DHA).

### Statistical analysis

All the statistical analyses were performed using the R software (version 3.2.5) [32]. When needed, data were transformed in order to meet assumptions of variance analysis. A logarithmic transformation was used for mean body weights and a logit transformation was used for proportions (survival rate, composition of whole fish, nutrient retention and FA profile). For each parameter, the effect of the diet, the selection and the interaction between diet and selection were assessed using an analysis of variance. If the analysis of variance revealed a significant effect (cut-off p-value<0.01), a Tukey’s range test was also performed in order to detect which groups differed from each other (cut-off p-value<0.01).

Finally, for body weight and survival, the genetic gain for each diet (gain_*V*_ and gain_*M*_) which is the average trait improvement of selected fish in comparison to the control, were calculated to evaluate the efficiency of the selection, as follows:

$$\begin{array}{c} Gain_{V}=100*\frac{X_{SU-V}-X_{SY-V}}{X_{SY-V}} \end{array}$$ (6)

$$\begin{array}{c} Gain_{M}=100*\frac{X_{SUM}-X_{SY-M}}{X_{SY-M}} \end{array}$$ (7)

With Gain_*V*_, the gain when fish were fed with the V diet and Gain_*M*_ when fish were fed with the M diet. Positive genetic gains reflect higher performance in the selected fish, and thus confirmed the efficiency of the selection. In the case where selected fish perform better than the control, irrespective of the diet (fed the M or V diet), it is essential to calculate the specific gain for the V diet (specific V gain), as follows:

$$\begin{array}{c} Gain_{V_{Specific}}=Gain_{V}-Gain_{M} \end{array}$$ (8)

A positive specific V gain reveals a higher genetic gain when fish were fed the V diet than when fed the M diet. The higher the gain, the higher the selection is specific to the V diet. A positive interaction between the effect of diet and the effect of the selection is also used as an indicator of the selection specificity, as it reveals a significant difference between the genetic gain obtained for fish fed the M and the V diets.

### Ethical statement

Experimentation was conducted in the INRA experimental facilities (Peima facilities, Sizun, France and UMR Numéa, St-Pée-sur-Nivelle, France) authorized for animal experimentation by the French veterinary service which is the competent authority (B 29-277-02 and A 64-495-1). The experiments were in strict accordance with EU legal frameworks related to the protection of animals used for scientific research (Directive 2010/63/EU) and according to the National Guidelines for Animal Care of the French Ministry of Research (decree n°2013-118, february 1st, 2013). The scientists in charge of the experimentation received training and personal authorization (N° B64 10 003 and A29 102).

In agreement with ethical comittees “Comité d’Ethique Aquitaine Poissons Oiseaux” (C2EA-73) and “Comité d’Ethique Finisterien en Expérimentation Animale” (C2EA-74), the experiment reported here does not need approval by a specific ethical committee since it implies only classical rearing practices with all diets used in the experiment formulated to cover the nutritional requirements of Rainbow trout [33]. During the experiment, fish were daily monitored. If any clinical symptoms (i.e. morphological abnormality, restlessness or uncoordinated movements) were observed, fish were sedated by immersion in 2% benzocaine solution and then euthanized by immersion in a 6% benzocaine solution (anesthetic overdose) during 3 minutes.

## Results

### Mean body weights and survival rate before the 1st feeding

Egg weight (0.07±0.0 at 0 dpf) was not significantly different between the two lines SU and SY (P-value>0.05). Concerning survival, from hatching time to first feeding (41 dpf), there was a small but significant effect of the selection. Fish from the selected lines SU had a significantly lower survival rate than fish from the control SY line (96.3±0.17% and 98.5±0.15%, respectively, p-value<0.001).

### Growth performance and survival rate during experiment

**Fig 1:**
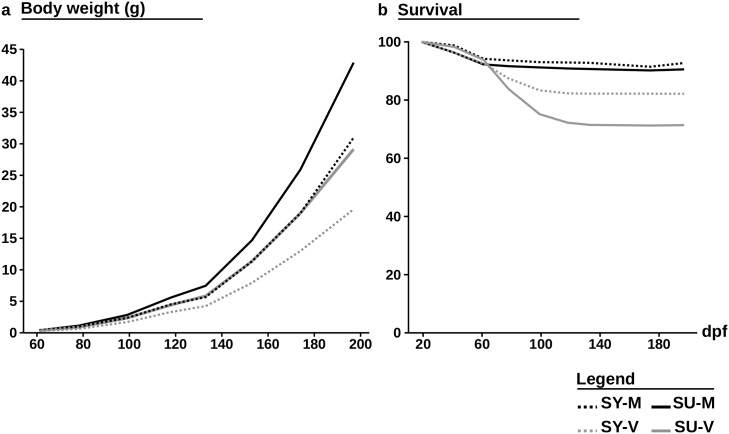
Growth and survival performance. (a) Body weights and (b) survival rate of the control (SY) and selected (SU) fish fed the M diet or the V diet (SY-M, SU-M, SY-V, SY-M) obtained for the period 1 and period 2.

After 7 months of feeding, fish reached a body weight of 42.9±1.0g for SU-M, 31.0±0.2g for SY-M, 29.4±0.6g for SU-V and 19.6±0.21g for SY-V. Body weights and survival rates obtained during the experiment are presented in Fig 1. Results of the statistical analysis on mean body weight are presented in Table 3. At each date, there was an effect of both the diet and the selection. While, the V diet diminished fish body weight, the selection increased it. Interestingly, there were no significant differences between the mean body weight of SU-V fish and SY-M fish from 78 dpf until the end of the trial.

**Table 3:** Results of the statistical analysis on mean body weight.

	SY-M	SU-M	SY-V	SU-V	Statistical analysis		
					Diet	Selection	Diet×Selection
**Period 1 from 41 to 153 dpf**							
**61**	0.4±0.0ab	0.4±0.0a	0.3±0.0c	0.4±0.0b	<0.001	<0.001	<0.001
**78**	1.0±0.0ab	1.1±0.0a	0.6±0.0c	0.9±0.0b	<0.001	<0.001	<0.01
**99**	2.4±0.0b	2.8±0.0a	1.7±0.0c	2.3±0.0b	<0.001	<0.001	<0.01
**118**	4.5±0.0b	5.6±0.1a	3.3±0.1c	4.4±0.1b	<0.001	<0.001	*ns*
**133**	5.7±0.1b	7.5±0.1a	4.2±0.1c	5.8±0.1b	<0.001	<0.001	*ns*
**153**	11.3±0.2b	14.7±0.2a	7.9±0.1c	11.3±0.2b	<0.001	<0.001	<0.01
**Period 2 from 153 to 197 dpf**							
**174**	19.0±0.1b	25.9±0.5a	13.0±0.3c	19.0±0.4b	<0.001	<0.001	*ns*
**197**	31.0±0.2b	42.9±1.0a	19.6±0.2c	29.1±0.6b	<0.001	<0.001	*ns*

Mean body weight in g (mean±standard error) of the control (SY) and selected (SU) fish fed the M diet or the V diet (SY-M, SU-M, SY-V, SY-M) during the experiment and their statistical analyses for the effects of the diet, of the selection and of the interaction between diet and selection (cutoff P-value<0.01). Different letters indicate significant differences between groups assessed with a post-hoc test.

**Fig 2:**
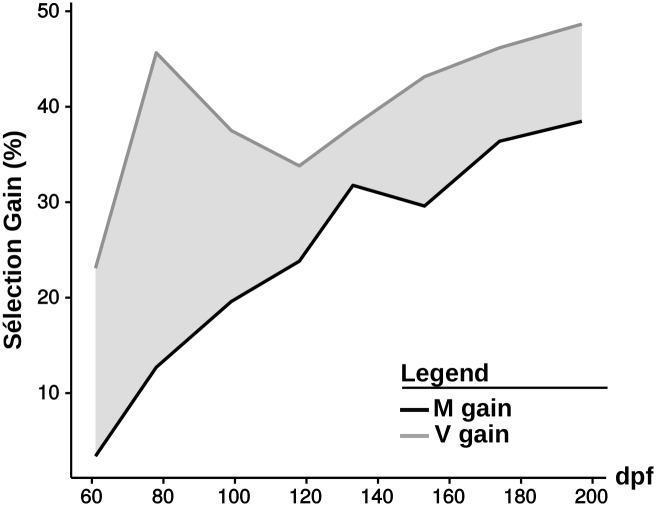
Selection gain obtained after three generation of selection. Selection gain obtained for mean body weights (%) for fish fed with the V diet (green line) and fish fed the M diet (blue line). The difference between the two curves represent the specific gain for plant-based diet.

The positive M and V genetic gains obtained for body weight (Fig 2) reflect the efficiency of the selection throughout the experiment. The specific V gain increased from 19.7% to 33.0% at 78 dpf and then decreased to 10.2% (197 dpf). This positive result, along with the significant interaction between diet and selection (61, 78, 99 and 153 dpf) reflect the specificity of the selection.

Results of the statistical analysis on survival are presented in Table 4. After first feeding, survival rate of fish fed the V diet dropped significantly and finally stabilized at 82.2±3.3% for SU-V and 71.4±2.7% for SY-V at 197 dpf, while the survival rate for fish fed the M diet remained stable (SU-M = 90.5±0.3%, SY-M = 92.7±0.3% at 197 dpf). From 78 dpf, there was a strong effect of the diet. A positive interaction between the selection and the diet appeared at 118 dpf, and reveal the efficiency and the specificity of the selection. Post-hoc tests revealed that SU-V had a significant higher survival rate than SY-V (P-values<0.01), while no differences were recorded between SY-M and SU-M fish (P-values>0.05).

**Table 4:** Results of the statistical analysis on survival.

	SY-M	SU-M	SY-V	SU-V	Statistical analysis		
					Diet	Selection	Diet×Selection
**Period 1 from 41 to 153 dpf**							
**41**	98.8±0.2a	96.3±0.1b	98.3±0.2a	96.3±0.4b	-	<0.001	-
**61**	94.2±0.3	92.2±0.7	93.8±0.8	92.6±0.6	*ns*	*ns*	*ns*
**78**	93.6±0.4a	91.6±0.5ab	83.8±1.9c	87.4±1.7bc	<0.001	*ns*	*ns*
**99**	93.0±0.3a	91.2±0.5a	75.2±2.6b	83.3±2.9b	<0.001	*ns*	*ns*
**118**	92.9±0.3a	90.8±0.4a	72.2±2.8c	82.3±3.2b	<0.001	*ns*	<0.01
**133**	92.8±0.3a	90.7±0.3a	71.5±2.7 c	82.2±3.3b	<0.001	*ns*	<0.01
**153**	92.7±0.3a	90.5±0.3a	71.4±2.7 c	82.2±3.3b	<0.001	*ns*	<0.01
**Period 2 from 153 to 197 dpf**							
**174**	92.7±0.3a	90.5±0.3a	71.4±2.7c	82.2±3.3b	<0.001	*ns*	<0.01
**197**	92.7±0.3a	90.5±0.3a	71.4±2.7c	82.2±3.3b	<0.001	*ns*	<0.01

Survival (mean±standard error) of control (SY) and selected (SU) fish fed the M diet or the V diet (SY-M, SU-M, SY-V, SY-M) during the experiment and their statistical analyses for the effects of the diet, of the selection and of the interaction between diet and selection (cutoff P-value<0.01). Different letters mean significant differences between groups assessed with a post-hoc test.

### Feed intake, feed efficiency

**Fig 3:**
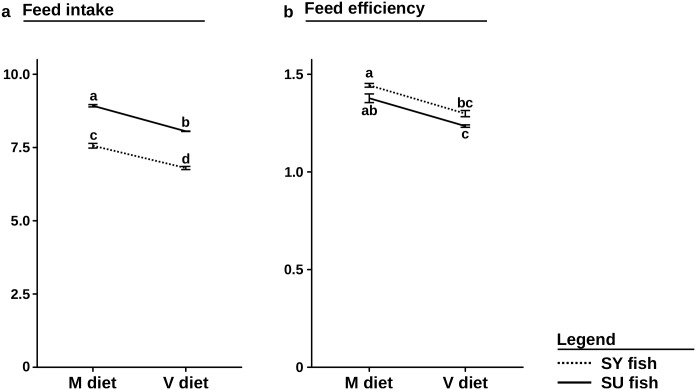
Results of the statistical analysis on feed intake and feed efficiency. (a) Mean feed intake and (b) feed efficiency (mean ± standard error) estimated during the second period of 44 days (from 153 to 197 dpf) for control and selected fish fed the M diet or the V diet (SU-M, SU-V, SY-M, SY-V). Different letters mean significant differences (P-value<0.01) between the four groups.

The quantity of feed ingested for each batch was measured precisely during a 44 day period, which allowed us to estimate the feed intake and then the feed efficiency per batch (Fig 3). Concerning both the feed intake and the feed efficiency, there was a significant effect of the diet (P-value<0.001) and the selection (P-values<0.001), but no significant interaction. The V diet significantly lowered the feed intake, while the selection significantly increased it. Interestingly, the SU-V fish had a higher feed intake than the SY-M fish (P-value<0.01). Similarly, the V diet and the selection significantly lowered the feed efficiency.

### Nutrient apparent digestibility

Apparent digestibility coefficients (ADC) and the associated statistical results are presented in Table 5. For each nutrient, there was a strong diet effect. The V diet significantly decreased the ADC of moisture, lipid, energy and starch. In contrast, the ADC for proteins was higher in fish fed the V diet. Concerning lipids, there was a significant effect of the selection. However, differences were small and post-hoc testing did not detect any differences between SY-M and SU-M (P-value>0.01); nor between SY-V and SU-V (P-value>0.01). For starch, there was a significant effect of the selection and an interaction. SU fish had significantly higher starch ADC values when fed the M diet (P-value<0.001), but no significant differences were detected when fish were fed the V diet (P-value = 0.13).

**Table 5:** Results of the statistical analysis on apparent digestibility.

	SY-M	SU-M	SY-V	SU-V	Statistical analysis		
					Diet	Selection	Diet×Selection
Moisture	83.5±0.0a	83.9±0.3a	81.4±0.1b	81.7±0.2b	<0.001	*ns*	*ns*
Lipid	97.2±0.1a	97.4±0.0a	95.3±0.0b	95.8±0.0b	<0.001	<0.01	*ns*
Protein	91.5±0.1b	91.2±0.3b	96.7±0.1a	96.8±0.1a	<0.001	*ns*	*ns*
Energy	91.4±0.1a	91.7±0.2a	88.4±0.2b	88.7±0.1b	<0.001	*ns*	*ns*
Starch	98.0±0.3b	99.3±0.0a	91.1±0.6c	93.5±0.4c	<0.001	<0.001	<0.01

Average apparent digestibility of nutrients and energy (%) (mean ± standard error) for control and selected fish fed the M diet or the V diet (SU-M, SU-V, SY-M, SY-V) during 23 days and their statistical analyses for the effects of the diet, of the selection and of the interaction between diet and selection (cutoff P-value<0.01). Different letters indicate significant differences between groups assessed with a post-hoc test.

### Nutrient retention, composition of whole fish and fatty acid profile

Results of composition of whole body and nutrient retention are presented in Table 6. There was a significant diet effect. The V diet enhanced the whole lipid content and energy (at 153 dpf only). Selection had no significant effect on any of these parameters.

**Table 6:** Results of the statistical analysis on composition of whole fish and nutrient retention.

	SY-M	SU-M	SY-V	SU-V	Statistical analysis		
					Diet	Selection	Diet×Selection
**Composition of whole fish at 153 dpf**							
Moisture	73.5±0.3	73.4±0.1	72.8±0.4	72.4±0.2	*ns*	*ns*	*ns*
Lipid	2.9±0.1b	2.9±0.0b	3.1±0.1ab	3.4±0.1a	<0.001	*ns*	*ns*
Protein	13.9±0.1	13.8±0.1	14.0±0.2	13.7±0.1	*ns*	*ns*	*ns*
Energy	6.8±0.1b	6.7±0.0b	7.1±0.1ab	7.5±0.1a	<0.001	*ns*	*ns*
**Composition of whole fish at 197 dpf**							
Moisture	71.8±0.2	70.7±0.3	70.9±0.6	69.8±0.1	*ns*	*ns*	*ns*
Lipid	3.3±0.1b	3.6±0.1ab	4.0±0.2a	4.1±0.1a	<0.001	*ns*	*ns*
Protein	15.5±0.1	15.6±0.1	15.2±0.1	15.2±0.1	*ns*	*ns*	*ns*
Energy	7.2±0.1	7.7±0.1	7.6±0.2	8.0±0.1	*ns*	*ns*	*ns*
**Nutrient retention (% Digestible intake)**							
Lipid	27.3±0.9	29.4±0.6	33.4±2.2	31.4±0.4	*ns*	*ns*	*ns*
Protein	51.8±0.2a	49.6±0.6a	41.7±0.6b	40.3±0.4 b	<0.001	<0.01	*ns*
Energy	52.0±0.3	54.0±0.5	49.4±2.2	49.2±0.3	*ns*	*ns*	*ns*

Average initial (at 153 dpf) and final (at 197 dpf) composition of whole fish (%) and nutrient retention (%) (mean ± standard error) for control and selected fish fed the M diet or the V diet (SU-M, SU-V, SY-M, SY-V) and their statistical analyses for the effects of the diet, of the selection and of the interaction between diet and selection (cutoff P-value<0.01). Different letters indicate significant differences between groups assessed with a post-hoc test.

Both the diet and the selection affected the efficiency of protein retention. Fish fed the V diet retained less protein than fish fed the M diet (P-value<0.001). And on average, the selection negatively affected the protein retention of the SU fish. However further testing did not reveal any differences between SY-M and SU-M (P-value>0.01), or between SY-V and SU-V (P-value>0.01).

Finally, the V diet highly affected the fatty acid profile at both time points (P-values<0.001, Table 7). While the V diet decreased the overall proportion of saturated FA and n-3 PUFA, the overall proportion of mono-unsaturated fatty acid (MUFA) and n-6 PUFA fatty acids was increased. Specifically, both the linoleic acid (LA) and the linolenic acid (ALA), the V diet increased their proportions.

**Table 7:** Results of the statistical analysis on FA profile.

	SY-M	SU-M	SY-V	SU-V	Statistical analysis		
					Diet	Selection	Diet×Selection
**Fatty acid profile at 153 dpf**							
**Σ Saturated**	35.9±0.7a	38.9±0.5a	22.0±0.4b	22.3±0.9b	<0.001	*ns*	*ns*
**Σ MUFA**	38.5±0.1b	38.1±0.2b	42.8±0.2a	42.5±0.2a	<0.001	*ns*	*ns*
**Σ n-6 PUFA**	7.2±0.1b	7.2±0.1b	22.5±0.1a	22.3±0.4a	<0.001	*ns*	*ns*
→18:2 n-6 (LA)	2.7±0.0b	2.6±0.0b	18.8±0.0a	18.8±0.3a	<0.001	*ns*	*ns*
**Σ n-3 PUFA**	15.0±0.7	12.3±0.5	12.2±0.2	12.3±0.6	*ns*	*ns*	*ns*
→18:3 n-3 (ALA)	0.6±0.0b	0.6±0.0b	7.3±0.1a	7.6±0.2a	<0.001	*ns*	*ns*
→20:5 n-3 (EPA)	5.8±0.2a	5.0±0.2a	0.5±0.0b	0.5±0.1b	<0.001	*ns*	*ns*
→22:6 n-3 (DHA)	5.6±0.4a	4.2±0.2a	1.0±0.1b	1.1±0.2b	<0.001	*ns*	*ns*
**Fatty acid profile at 197 dpf**							
**Σ Saturated**	37.2±1.1a	39.5±0.4a	21.8±0.5b	23.3±0.2b	<0.001	*ns*	*ns*
**Σ MUFA**	35.6±0.5b	36.0±0.7b	44.0±0.2a	44.8±0.8a	<0.001	*ns*	*ns*
**Σ n-6 PUFA**	7.5±0.2b	7.3±0.1b	22.0±0.1a	21.0± 0.3a	<0.001	*ns*	*ns*
→18:2 n-6 (LA)	3.1± 0.1b	3.0±0.0b	18.8±0.1a	18.3± 0.2a	<0.001	*ns*	*ns*
**Σ n-3 PUFA**	16.4±0.9a	14.3±0.3ab	11.8± 0.2c	10.5±0.5bc	<0.001	*ns*	*ns*
→18:3 n-3 (ALA)	0.8± 0.0b	0.8±0.0b	7.8±0.2a	7.4±0.3a	<0.001	*ns*	*ns*
→20:5 n-3 (EPA)	5.7± 0.1a	5.2±0.0a	0.4± 0.0b	0.3± 0.0c	<0.001	<0.01	*ns*
→22:6 n-3 (DHA)	6.9±0.7a	5.7±0.2a	0.8±0.1b	0.6±0.0b	<0.001	<0.01	*ns*

Fatty acid composition (% of total FA) at 153 dpf and at 197 dpf (mean±standard error) for control and selected fish fed the M diet or the V diet (SU-M, SU-V, SY-M, SY-V) and their statistical analyses for the effects of the diet, of the selection and of the interaction between diet and selection (cutoff P-value<0.01). Different letters indicate significant differences between groups assessed with a post-hoc test.

Concerning EPA and DHA, the V diet significantly decreased their proportions on whole fish body content. The selection also affected their proportion at 197 dpf (P-values<0.01). For EPA, no differences were observed when fish were fed the M diet (P-value = 0.35) but SY-V fish tended to have a higher EPA proportion than SU-V (P-value = 0.01). A similar pattern was observed for DHA. While no differences were observed when fish were fed the M diet (P-value = 0.25), SY-V tended to have higher final DHA proportion than SU-V (P-value = 0.07).

When EPA and DHA were considered in term of absolute content in whole fish (Table 8), there was an effect of the diet (P-values<0.001), and an effect of the interaction for EPA at 153 dpf (P-values<0.001). While the V diet decreased the EPA and DHA amount in both lines, the SY line had a higher EPA content than that of the SU when fed the M diet (P-value<0.01) at 153 dpf.

**Table 8:** Results of the statistical analysis on EPA and DHA content.

	SY-M	SU-M	SY-V	SU-V	Statistical analysis		
					Diet	Selection	Diet×Selection
**At 153 dpf**							
20:5 n-3 (EPA)	168.3±5.1a	143.8±5.0b	14.2±1.1c	16.0±1.9c	<0.001	*ns*	<0.01
22:6 n-3 (DHA)	161.5±11.9a	120.1±5.1a	32.4±3.7b	36.4±7.7b	<0.001	*ns*	*ns*
**At 197 dpf**							
20:5 n-3 (EPA)	188.7±3.4a	189.2±0.6a	14.8±0.5b	12.2±0.8b	<0.001	*ns*	*ns*
22:6 n-3 (DHA)	228.9±22.6a	206.8±8.6a	32.9±2.1b	25.5±1.2b	<0.001	*ns*	*ns*

EPA and DHA content (mg/100g tissue) at 153 dpf and at 197 dpf (mean±standard error) for control and selected fish fed the M diet or the V diet (SU-M, SU-V, SY-M, SY-V) and their statistical analyses for the effects of the diet, of the selection and of the interaction between diet and selection (cutoff P-value<0.01). Different letters indicate significant differences between groups assessed with a post-hoc test.

Responses of all the nutritional traits affected by the V diet and/or the selection are summarized in Table 9.

**Table 9:** Summary of the different nutritional traits affected by the plant-based diet and by the selection (in bold).

	Effect of the V dietfor each line		Effect of the selection for each diet	
	SY-V *vs* SY-M	SU-M *vs* SU-V	SU-M *vs* SY-M	SU-V *vs* SY-V
**Final BW**	- 36.8%	- 32.2%	**+ 38.4%**	**+ 48.5%**
**Survival**	- 23.0%	- 9.2%	*ns*	**+ 15.2%**
**Feed intake**	- 10.5%	- 9.0%	**+ 17.1%**	**+ 19.1%**
**Feed efficiency**	- 7.1%	- 14.3%	0.0%	**-7.7%**
**Digestibility**				
→ Protein digestibility	+ 5.7%	+ 6.1%	*ns*	*ns*
→ Starch digestibility	- 7.0%	- 5.8%	**+ 1.3%**	*ns*
→ Lipid digestibility	- 2.0%	- 1.6%	*ns*	**+ 0.5%**
**Retention**				
→ Lipid retention	+ 22.3%	+ 6.8%	*ns*	*ns*
→ Protein retention	- 19.5%	- 18.8%	**- 4.2%**	*ns*
**FA Profile (197 dpf)**				
→ Lipid content	+ 21.2%	+ 13.9%	*ns*	*ns*
→ Saturated FA	- 41.4%	- 41.0%	*ns*	*ns*
→ MUFA	+ 23.6%	+ 24.4%	*ns*	*ns*
→ n-6 PUFA	+ 193.3%	+ 187.7%	*ns*	*ns*
→ n-3 PUFA	- 28.1%	- 26.6%	*ns*	*ns*
→ EPA	-93.0%	- 94.0%	*ns*	**- 25.0%**
→ DHA	-88.4%	- 89.5%	*ns*	**- 25.0%**
**EPA and DHA content (153 dpf)**				
→ EPA (mg/100g tissue)	- 91.6%	- 88.9%	**- 14.6%**	*ns*
→ DHA (mg/100g tissue)	- 79.9%	- 69.7%	**- 25.6%**	*ns*

## Discussion

In the current context where aquaculture reliance on marine products must be reduced, we have selected a line of rainbow trout for its ability to survive and grow with a 100% plant-based diet from the first feeding. We will first discuss the effect of such an extreme diet completely devoid of marine products, then the efficiency of the selection and its indirect consequences.

### Incorporation of VO and VM affects fish performance

The present results confirmed that rainbow trout growth and survival are highly affected when fed a 100% plant-based diet since the early first-feeding, as it was previously shown [9, 10]. The majority of the nutritional traits studied were highly impaired by the V diet, in both lines. This decreased growth performance was first associated with a reduced feed intake (-9.0% for SU and -10.5% for SY line) in accordance with previous studies on plant-based diet replacement [34–36]. Causes of feed intake reduction with a plant-based diet is not well understood in fish and could be linked to either a change in feed palatability or a negative feedback due to the nutritional quality of the V diet [37]. Second, feed efficiency was reduced for both SU-V (-14.3%) and SY-V (-7.1%) as reported before with a plant-based diet [38]. Reduced feed efficiency may stem from reduced nutrient digestibility. Both starch and lipid digestibility were reduced with the V diet, thus explaining the lower digestibility for energy. In contrast, protein digestibility was high and in line with known values as the V diet was formulated with highly digestible protein concentrate [33, 34]. Unknown nutrient deficiencies could also lead to decreased performance of fish fed a plant-based diet [33]. Although the experimental V diet was formulated to meet known nutrient requirements for rainbow trout, the large reduction in protein retention when fish were fed the V diet (-19.5% for SY fish, and -18.8% for SU fish) suggests an amino acid imbalance. Finally, anti-nutritional factors (ANF) present in plant-based diets are known to be one of the major factors affecting nutrient utilization [13–15] and could be a cause for these decreases in performance. Despite being formulated with a blend of vegetable meals followed by extrusion to avoid the effects of such components, some remaining ANFs could have affected fish capacity to properly use nutrients.

Whole body composition and whole FA profile were also highly affected by the V diet for both SU and SY lines. Fish fed the V diet, irrespective of the lines, had higher fat content than fish fed the M diet, as it was previously observed when rainbow trout were fed plant-based diets [28, 39, 40]. Those differences are linked with higher lipid retention observed in fish fed the V diet, although differences were not significant due to the high heterogeneity in SY-V group. Finally, both SY-V and SU-V FA profiles were highly impaired, and reflected the FA profile of the V diet as previously shown in rainbow trout [41] and in salmon [42–44]. However, significant amounts of EPA and DHA were found in fish whole body, even though fish were fed with a diet completely devoid of these two FA since their first feeding, confirming previous results which demonstrated the ability of rainbow trout to neo-synthetise EPA and DHA from ALA [45].

### Selection improves survival and growth

The major finding of the present study is that after only 3 generations of selection, selected fish fed the V diet (SU-V) reached a similar final body weight as the control fish fed the marine resources-based diet (SY-M), along with an improved survival at early stage (Fig 1). These results are very promising as only three generations were needed to overcome the major detrimental effect of the V diet on growth (- 36.8% in the SY line at 197 dpf). Yamamoto *et al.* found a similar result with amago salmon selected for their ability to use a fishmeal-free diet, which reached the same body weight as control fish fed a marine resources-based diet after 3 generations of selection. In contrast to other terrestrially farmed animals, genetic gains obtained per generation in fish are expected to be large thanks to high fecundity, and an important genetic and phenotypic variation [46]. This was the case in different breeding programs where rainbow trout were selected for rapid growth [47–50].

An analysis of the observed genetic gains allows for the efficiency and specificity of the selection to be evaluated. Interestingly, the gain obtained for body weight varied within the experiment and could be explained by the long time it takes for the SY-V fish to die. At the beginning of the trial, a portion of the control fish that were fed the V diet (SY-V) did not accept the V diet and remained small, thus increasing the disparities between the mean body weight of the two lines fed the V diet, explaining the observed peak. Once these fasting SY-V fish started to die, the disparity between mean body weights among the different groups narrowed, reducing the specific V gain.

Nevertheless, the genetic gain obtained for the V diet confirmed the results recorded after the first generation of selection [27] and confirmed the efficiency of the selection for both body weight and survival with the V diet devoid of marine resources. While the selection was really specific to the V diet for the survival, the specificity was inferior for the body weight as SU-M body weights were also improved. These results are in line with what has been observed after the first generation of selection [27] but differ from those obtained by Overturf *et al.* [23] where rainbow trout was selected on the ability to use a fishmeal-free diet. In this latter study, the growth performance of the selected fish fed the standard diet was also improved, but selected fish fed the plant-based diet reached a higher body weight than the selected fish fed the standard diet. The two experimental diets used for the selection were extremely different. Our diet was completely devoid of both FM and FO, and was thus more extreme. Due to these differences, the selection seemed to have improved different traits between Overturf’s study and ours.

Lastly, looking at the evolution of the genetic gain over generations allows the gains obtained between the first generation of selection [27] and the third to be compared. Concerning body weight, the genetic gain obtained with the V diet averaged 30.4% in the first generation (from 60 to 193 dpf) and 39.0% in the third one (from 41 to 197 dpf, removing the peak at 73 dpf). Concerning survival, the genetic gain obtained with the V diet was equal to 16.2% at 193 dpf in the first generation of selection and 15.1% at 197 dpf in the third. These results could indicate a limited improvement for survival and a slower one for body weight made since the first generation.

**Fig 4:**
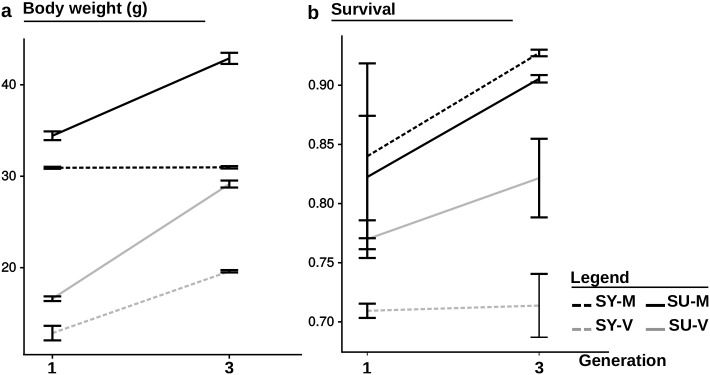
Performance comparison between the first and third generation of selection. Evolution of (a) mean body weights and (b) survival between the first and the third generation of selection for the 4 different conditions (SU-M, SY-M, SU-V and SY-V), at 193 dpf for the first generation, and 197 dpf for the third one.

However, these results should be interpreted cautiously as the absolute growth performance of the control line fed the V diet (SY-V) was higher during the third generation than it was in the first (Fig 4). Inconsistent performance between generations in the control line (SY) could be attributed to environmental cues, as has been demonstrated in seabass which are very sensitive to their environment when fed a complete plant-based diet from a very young stage [51]. However, our data do not allow for an adequate assessment of the effect of environment on SU-V and SY-V. Further, it is possible that an interaction between environmental cues and the line (SU-V and SY-V) may also exist. Where the interaction between environmental cues and the genotype would have been null, the genetic gain would have been comparable. However, in the case of a significant interaction, genetic gain would have been either over-estimated (SU-V more positively affected) or under-estimated (SY-V more positively affected). Diet must be considered as a potential environmental factors, as the blend of vegetable meals used for the selection was modified between the second and the third generations (diet V-1 and diet V, see Table 1). In addition, the quality of raw products may vary over time, which could also impact the composition of the diet. To better define the problem, more data spanning several generations are necessary to fully understand if one or both lines fed the V diet were affected by the diet evolution or other environmental factors.

### High genetic progress linked to higher feed intake

To understand the increased growth performance of the selected line, feed intake and feed efficiency (the two main determinants of growth rate), were estimated during a 44-day feeding trial. We found that improved performance was related to a higher feed intake rather than to an improved feed efficiency (Fig 3). The selection significantly improved feed intake, but it was not specific to the V diet and SU fish ate on average 18.2% more than SY fish, irrespective of the diet. This result explains the general genetic gain observed for both SU-M and SU-V, which could suggest a selection on fish with a higher eating capacity. Surprisingly, selected fish fed the V diet ate significantly more than control fish fed the M diet (SU-M), and it cannot be excluded that the selection has also improved fish resilience to plant components. These results support the findings of Yamamoto *et al.* who showed that increased fish performance was attributed to a higher feed intake, irrespective of the diet [24–26]. This result highlights the fact that acceptance of the plant-based diet is a major step impeding the replacement of fish oil and fishmeal, and can be significantly improved by selection.

### Selection has indirect consequences on feed efficiency, starch digestibility, protein retention and EPA/DHA content

The observed trend toward a reduced feed efficiency in the SU line was opposite to what we would have expected. Yet, the selection seems to slightly improve the lipid and starch digestibility. The differences with respect to the digestibility of lipids seem too small to have had any biological impacts (+0.5%). The starch digestibility was significantly improved in SU fish when fed the M diet (+1.3%, P-value<0.001), but no difference was observed when fish were fed the V diet (P-value = 0.13). rainbow trout, like other carnivorous fish, digested starch poorly due to the low activity of the alpha-amylase [52]. However, experimental diets were formulated with gelatinized starch, and starch digestibility was already high. This result is thus quite surprising. Measurement of alpha-amylase activity between the two lines should be performed to determine if it is responsible for the improvement in the digestibility of starch.

In contrast, the selection negatively affected the overall protein retention and the SU-M tended to retain 4.2% less protein than SY-M (P-value = 0.06). Surprisingly protein retention was not affected when fish were fed the V diet (P-value = 0.24). In contrast, Overturf *et al.* found an improvement of protein retention in their selected line of rainbow trout in comparison with the control one after 4 generations of selection [23], linked in part to the improvement of ANFs tolerance [53]. In the present study, protein retention was affected when fish were fed the M diet, and thus the observed differences were not only a result of the potentially negative impact of ANFs on the intestine. Thus, the differences between the selected and the control lines are not yet explained.

Finally, the selection did not counteract the well-known effects of the V diet on the FA profile, nor the whole fish lipid content. But, the selection did negatively impact the proportion of EPA and DHA in the whole body of SU-V fish as well as the EPA content of SU-M fish. These two FA play essential roles and EPA or DHA deficiencies are known to affect growth in rainbow trout [54]. In addition to being used as a source of energy like other fatty acids, EPA is a precursor for eicosanoids, whereas DHA plays important structural and functional roles in cell membranes [55]. When fish are fed a plant-based diet, the use of EPA for energy production is usually restrained [56].

First, selection could have negatively affected SU fish EPA and DHA biosynthesis capacity, in contrast to what would have been expected as SU-V fish growth improved. Second, the selection could have impacted the level of EPA used for energy production. However, our experimental design does not make it possible to answer this question. EPA and DHA are beneficial for the human consumer, as these two FA play various important roles in human health [57]. A finishing period where fish are fed a marine resources-based diet are considered to restore EPA and DHA levels in fish [6]. It is thus essential to test whether the selection affected the capacity to biosynthesize these FA for both those fish fed the V diet as well as the classic marine resource-based diet. Further analyses are first needed after further generation of selection to confirm this result, and second to test both the expression and activity of elongase and desaturase, enzymes responsible for EPA and DHA synthesis and whole fish EPA and DHA content for fish at market size. Finally, these results highlight the importance to take into account n-3 PUFA profile during breeding program, as previous studies had shown that it is a highly heritable trait in salmon [58].

## Conclusion

The results obtained for the third generation rainbow trout selected for their growth on a V diet confirm that selection has successfully improved both the mean body weight and survival of the selected versus control fish fed the V diet. SY-V fish now reach the same final body weight as control fish fed the M diet.

In the present study, we were able to attribute the better growth performance of the selected line to a higher feed intake. This result confirms that acceptance of a plant-based diet, one of the major steps impeding the total replacement of marine ingredients, can be improved by selection [26]. The reluctance of fish to eat plant-diets remains poorly understood. Hence, the potential to lower sensitivity to plant-diet components through selection, leading to higher feed intake, requires further investigation. The two lines used in this study are a good models to work on.

Aside from feed intake, the selection had a limited effect on other nutritional traits in comparison with the diet effect. But the effect of the selection will increase with generations of selection, while the dietary effect should remain stable. It is therefore essential to carefully monitor its consequences such as the EPA/DHA biosynthesis capacity, but also its consequences on traits which were affected by the selection only when fish were fed the M diet (protein retention and starch digestibility).

Finally, the feed intake of the selected line was improved similarly when fish were fed the M or the V diets. Our study did not allow us to identify a specific nutritional trait that was significantly improved when fish were fed the V diet only, which could explain the higher genetic gain recorded for those fish. Other traits such as the immune parameters or the microbiota should also be explored in future generations as they are also known to influence the performance of fish fed a plant-based diet.

## Supporting information

S1 File ARRIVE guidelines checklist. (PDF)
